# An Upstream Open Reading Frame Regulates LST1 Expression during Monocyte Differentiation

**DOI:** 10.1371/journal.pone.0096245

**Published:** 2014-05-09

**Authors:** Christian Schiller, Carina Nowak, Kalliope N. Diakopoulos, Ulrich H. Weidle, Elisabeth H. Weiss

**Affiliations:** 1 Department of Biology II, Ludwig-Maximilians-Universität München, Germany; 2 Division Pharma, Roche Diagnostics, Penzberg, Germany; University of British Columbia, Canada

## Abstract

The regulation of gene expression depends on the interplay of multiple factors at the transcriptional and translational level. Upstream open reading frames (uORFs) play an important role as translational repressors of main ORFs and their presence or usage in transcripts can be regulated by different mechanisms. The main objective of the present study was to assess whether uORFs regulate the expression of the MHC class III gene *LST1*. We report that expression of *LST1* is tightly regulated by alternative transcription initiation and the presence of an uORF in the 5′-UTR of transcripts. Specifically, using EGFP reporter constructs in human HeLa and HEK-293T cells and flow cytometry as well as western blot analysis we found the uORF to reduce the expression of the main ORF by roughly two-thirds. Furthermore, we were able to correlate a previously detected increase in LST1 protein expression during monocyte differentiation with an increase of transcription initiation at an alternative exon that does not contain an uORF.

## Introduction

The regulation of gene expression at the translational level depends mainly on specific structures in the 5′ and 3′ untranslated regions (UTR) of mRNAs (for a review see [1]). Upstream open reading frames (uORF) are often found in the 5′-UTR of mammalian genes and can inhibit translation of the main ORF by recruitment and premature dissociation of the ribosome, which subsequently fails to initiate translation at the main ORF. The factors determining translational inhibition of the main ORF include: strength of the Kozak sequence at the uORF start codon, uORF length, uORF sequence and distance to main ORF (for a review see [2]). It has been estimated that 44–49% of all human genes display uORFs and their presence generally correlates with reduced protein expression [3], [4]. A similar percentage of mouse genes have been found to contain uORFs. However, both in the human and mouse transcriptomes the number of uORFs present is lower than statistically expected, indicating that they have undergone purifying selection [3]. Nevertheless, uORFs are overrepresented in genes whose expression is tightly regulated including transcription factors, growth factors, and their receptors [5], thereby pointing to an important role for uORFs in gene dose regulation. Furthermore, recent studies indicate that translational regulation by uORFs is an evolutionary conserved mechanism as it has also been detected in yeast and fungi [6], [7].

The *LST1* (*L*eukocyte *S*pecific *T*ranscript *1*) gene is localized in the HLA class III region on chromosome 6. Its primary transcript undergoes substantial alternative splicing, resulting in 17 splice variants that potentially encode 12 different protein isoforms. Depending on exon usage the resulting proteins are either short soluble molecules or transmembrane proteins. *LST1* encompasses 9 exons, transcription can be initiated at each of the 5 noncoding exons 1A–E [8], while translation always starts at a specific ATG on exon 2 [9]. LST1 proteins have been postulated to have an immunoregulatory function [10] and to play a role in NFκB regulation [11]. Additionally, we have recently found transmembrane LST1 to regulate cell-cell communication [12]. Several studies have proposed that *LST1* may play a role in inflammatory diseases including Reactive Arthritis [13], Lupus Nephritis [14] and the inflammatory response believed to cause Neural Tube Defect [15]. These studies correlated high levels of *LST1* expression with inflammatory pathologies, thereby further substantiating a potential link between LST1 protein levels and inflammation. However, it remains unclear whether LST1 protein expression is mainly regulated at the transcriptional or the translational level, or both. In the present study we have addressed the question whether LST1 protein levels are translationally regulated by differential usage of the noncoding exons 1A–E in transcripts, some of which contain uORFs.

## Materials and Methods

### Generation of expression constructs

The Exon1B-EGFP and Exon1C-EGFP fusion constructs were cloned by amplifying either the *LST1* exon 1B or exon 1C sequence together with the 5′-UTR of exon 2 from cDNA, using specific primers. Amplificates were inserted into the pEGFP-N1 vector (Clontech) upstream (*Bam* HI site) of the ATG codon. The mutated constructs Exon1B-AUGdel-EGFP and Exon1B-frameshift-EGFP were generated using the Phusion site-directed mutagenesis kit from Finnzymes, following the manufacturer's instructions. To obtain an expression vector for red fluorescent protein, the cDNA encoding mCherry [16] was inserted into the pcDNA 3.1 (+) vector (Invitrogen). An overview of the expression constructs used in this study is displayed in Figure S1.

### Cell culture and transfection procedures

HeLa (ATCC CCL-2.1), HEK-293T (ATCC CRL-11268), U-937 (ATTC CRL-1593.2) and THP-1 (ATTC TIB-202) cells were cultivated as recommended by ATCC. HeLa cells were transfected with Lipofectamine 2000 (Invitrogen), following the manufacturer's instructions and using a 1∶1 DNA/reagent ratio. HEK-293T cells were transfected using PEI (Sigma-Aldrich) with the DNA/reagent ratio 1∶4. In vitro transcribed RNA (ivt RNA) was produced as described previously [17] and tested for integrity on agarose gels. For each transfection 500 ng of ivt RNA were introduced into HeLa cells via electroporation using 0.2 cm cuvettes and an Electro Cell Manipulator 660 (BTX) set to 250 V, 600 µF and 13 Ω. Transfectants were analysed 24 hours after transfection.

### Flow cytometry and western blot analysis

Flow cytometry was performed either with a FACS Calibur or a LSR Fortessa cytometer (BD Biosciences). For each sample at least 10,000 cells were analysed in triplicates. Western blot analysis was performed as described before [18]; blot imaging and signal quantification were conducted using the Odyssey infrared imaging system (Li-Cor). For quantitative analysis, western blots were imaged at least three times using different excitation intensities. Mean values from these acquisitions were normalized for the amount of tubulin in the corresponding lanes. The monoclonal antibody against tubulin (WA-3) was a kind gift from M. Schliwa (Ludwig-Maximilians-Universität München, Germany). The GFP antibody (A6455) was obtained from Molecular Probes.

### RT-PCR and qPCR procedures

U-937 and THP-1 cells were either treated with 80 nM TPA (12-O-Tetradecanoylphorbol-13-Acetate, from CellSignaling) or medium for 72 h. RNA was isolated using the Trifast reagent (Peqlab) and tested for integrity on agarose gels. cDNA synthesis was performed using M-MuLV Reverse Transcriptase (New England Biolabs) and an oligo-dT primer. *LST1* transcripts containing either the exon 1B or 1C sequence were detected via PCR using the following primers: 5-LST1B (TGGCCAGTTTGGAGTCTGTC), 5-LST1C (GAAGCAGCTCTCCACACCAG) and 3-LST1-Ex2 (TAGGCGAAATGATCAGGGGC). *GAPDH* transcripts were amplified with the following primers: 5-GAPDH (GTCTTCACCACCATGGAGAAGGCT) and 3-GAPDH (CATGCCAGTGAGCTTCCCGTTCA). *EGFP* transcripts were amplified with the following primers: 5-EGFP (GGCGATGCCACCTACGGCAAGC) and 3-EGFP (GTCGTGCTGCTTCATGTGGTCGGG). Transcripts of the neomycin resistance gene were amplified with the following primers: 5-Neomycin (CACAACAGAC AATCGGCTGCTCTGATG) and 3-Neomycin (CGCTGCCTCGTCTTGCAGTTCATTC). Quantitative PCR with cDNA or mock-treated RNA as a negative control was performed using the FastStart Universal SYBR Green Master (Roche) and a CDX96 Real-Time Cycler (Bio-Rad). No significant signals were obtained for the negative controls. Relative transcript quantities were calculated using the CFX Manager software (version 3.0, Bio-Rad).

### Statistical analysis

Differences between groups were tested for significance by applying the Mann-Whitney-U-test, using the BrightStat software [19]. Differences were considered significant at p<0.05.

## Results

### The *LST1* 5′-UTR contains several uORFs

**Figure 1:**
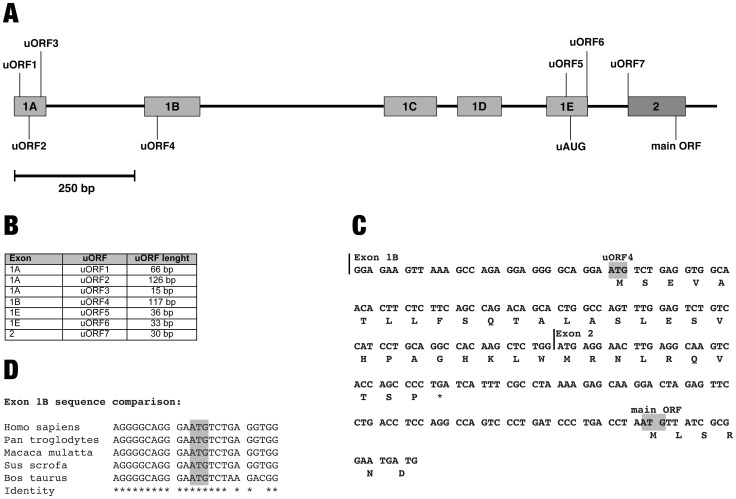
The *LST1* 5′-UTR contains several uORFs. **(A)** Schematic overview of the *LST1* 5′ exons. All *LST1* transcripts contain one of the noncoding exons 1A–E, which include transcription initiation sites, followed by the exon 2 sequence, which contains the start codon of the main ORF. The five alternative exons 1A–E and the exon 2 sequence are displayed as grey boxes; introns are represented by black lines. The start codons of uORFs 1–7 are indicated; additionally an uAUG in exon 1E is annotated. **(B)** List of uORFs in the *LST1* 5′-UTR and their corresponding lengths in base pairs from start to stop codon. **(C)** Sequence of the *LST1* 5′-UTR in transcripts initiated at exon 1B. The exon 1B and exon 2 sequences are labelled; the AUGs of uORF4 and of the main ORF are highlighted in grey. The amino acids encoded are indicated beneath the nucleic acid sequence. **(D)** Sequence comparison of the exon 1B sequence flanking the start codon of uORF4. The sequence is conserved in pan troglodytes (sequence accession number NW 003457113.1), macaca mulatta (NW 001116486.1), sus scrofa (NW 003610614.1) and bos taurus (NW 003104557.1).

All *LST1* transcripts contain one of the noncoding exons 1A–E followed by the exon 2 sequence, which contains the start codon of the main ORF. Several of the exon 1 variants contain uORFs of different lengths (Figure 1A–B). In the present study we focussed on the uORF of exon 1B (uORF4, Figure 1A) for several reasons. First, exon 1B is the most widely used exon 1 variant in *LST1*: roughly two thirds of all transcripts include the exon 1B sequence [8]. Second, the stop codon of uORF4 is located only 66 bp upstream of the main ORF and its last codon encodes proline (Figure 1C), a feature that has been shown to delay termination and therefore inhibit binding of other ribosomes to downstream ORFs in close proximity [20], [21]. Third, uORF4 is the only uORF in the 5′-UTR of *LST1* that is evolutionary conserved (Figure 1D and S1). Fourth, uORF4 is considerably longer than the average human uORF (51.5 bp; [3]) and the efficiency of translational inhibition has been described to increase with uORF length [22].

### The *LST1* exon 1B sequence inhibits protein expression

**Figure 2:**
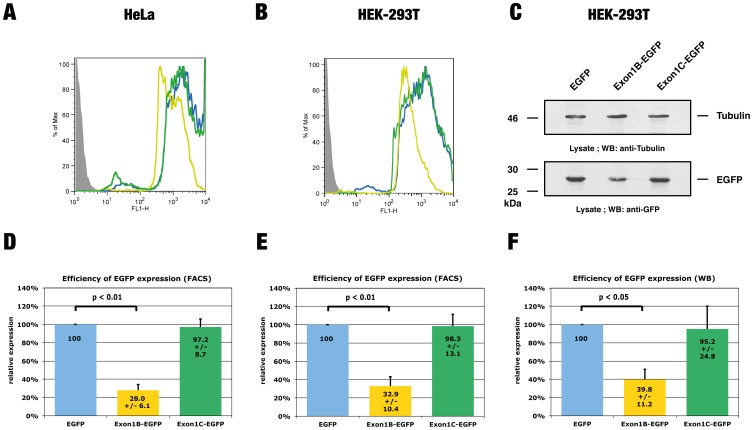
The *LST1* exon 1B sequence inhibits protein expression. HeLa and HEK-293T cells were transiently cotransfected with expression constructs encoding the red fluorescent protein mCherry and either an EGFP expression vector, Exon1B-EGFP or Exon 1C-EGFP fusion construct. **(A, B)** Flow cytometry analysis of EGFP intensity in HeLa (A) and HEK-293T (B) transfectants expressing Exon1B-EGFP (yellow line), Exon 1C-EGFP (green line) or the unmodified EGFP vector (blue line). Untransfected cells are displayed as a solid grey curve. The mCherry expression was used to gate and select positive transfectants, which were analysed for the intensity of EGFP expression. **(C)** Lysates from HEK-293T transfectants were probed by western blot analysis using a GFP-specific antibody (lower panel). To ensure that comparable amounts of protein were loaded, the membrane was additionally probed with a tubulin-specific antibody (upper panel). **(D, E)** Quantitative flow cytometry analysis of EGFP expression in HeLa (D) and HEK-293T (E) transfectants. The analysis of EGFP expression was performed as described in (A, B) and the mean fluorescence intensity was quantified. A value of 100% was set for cells transfected with the unmodified EGFP vector. Mean values from 5 independent experiments are indicated within the columns +/− s.d. Both HeLa and HEK-293T transfectants expressing Exon1B-EGFP displayed significantly reduced EGFP levels when compared with cells transfected with the empty vector (p = 0.009). **(F)** Quantitative western analysis of EGFP expression. Lysates were probed as described in (C). The EGFP signal intensity was quantified and normalized for tubulin expression. A value of 100% was set for lysates from cells transfected with the unmodified EGFP vector. Mean values from 3 independent experiments are indicated within the columns +/− s.d. Transfectants expressing Exon1B-EGFP displayed significantly reduced EGFP expression levels when compared with cells transfected with the empty vector (p = 0.049).

To ascertain whether the uORF4 of exon 1B regulates *LST1* protein expression, we generated reporter constructs containing the *LST1* 5′-UTR. These constructs included either the exon 1B or 1C sequence followed by the untranslated sequence of exon 2 and cloned upstream of the ORF encoding EGFP (Figure S2A - B). Transient expression of these constructs in HeLa cells, followed by flow cytometry analysis revealed that the exon 1B sequence significantly reduced the efficiency of EGFP protein expression, while the exon 1C sequence had no effect (Figure 2A and D). Similar results were obtained when EGFP expression was measured in HEK-293T transfectants via flow cytometry (Figure 2B and E) and quantitative western blot analysis (Figure 2C and F). Furthermore, the exon 1B sequence also displayed an inhibitory effect on EGFP expression, when we directly transfected HeLa cells with exon 1B-containing RNA, which was generated in vitro (Figure S3). The *LST1* exon 1C does not contain an uORF; the consistent downregulation of EGFP expression caused by the exon 1B sequence therefore indicates that the uORF4 of exon 1B may be responsible for the negative effect on protein expression.

### The uORF of *LST1* exon 1B inhibits protein expression

**Figure 3:**
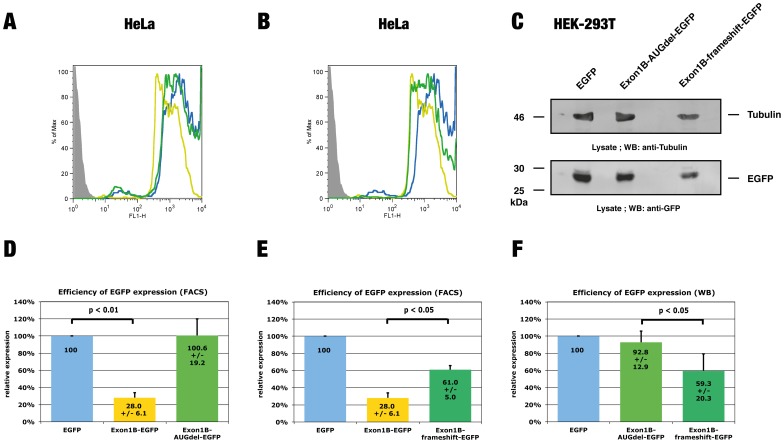
The uORF in *LST1* exon 1B inhibits protein expression. Cells were transiently cotransfected with expression constructs encoding the red fluorescent protein mCherry and either an EGFP expression vector, Exon1B-EGFP, Exon1B-AUGdel-EGFP or Exon1B-frameshift-EGFP fusion construct. **(A, B)** Flow cytometry analysis of EGFP intensity in HeLa transfectants expressing Exon1B-EGFP (yellow line), Exon1B-AUGdel-EGFP, Exon1B-frameshift-EGFP (green line in A and B, respectively) or the unmodified EGFP vector (blue line). Untransfected cells are displayed as a solid grey curve. The mCherry expression was used to gate and select positive transfectants, which were analysed for the intensity of EGFP expression. **(C)** Lysates from HEK-293T transfectants were probed by western blot analysis using a GFP-specific antibody (lower panel). To ensure that comparable amounts of protein were loaded, the membrane was additionally probed with a tubulin-specific antibody (upper panel). **(D, E)** Quantitative flow cytometry analysis of EGFP expression in HeLa transfectants. The analysis of EGFP expression was performed as described in (A, B) and the mean fluorescence intensity was quantified. A value of 100% was set for cells transfected with the unmodified EGFP vector. Mean values from 5 independent experiments are indicated within the columns +/− s.d. Transfectants expressing Exon1B-EGFP displayed significantly reduced EGFP expression levels when compared with cells transfected with the empty vector (p = 0.009). The expression of the Exon1B-AUGdel-EGFP vector, in which the start codon of uORF4 was deleted, was comparable to the expression of the unmodified construct. Expression of the Exon1B-frameshift-EGFP construct, in which a frameshift mutation shortens the length of uORF4, was significantly stronger when compared to cells transfected with the Exon1B-EGFP vector (p = 0.049), but still considerably weaker than the expression of the unmodified vector. **(F)** Quantitative western analysis of EGFP expression. Lysates were probed as described in (C). The EGFP signal intensity was quantified and normalized for tubulin expression. A value of 100% was set for lysates from cells transfected with the unmodified EGFP vector. Mean values from 3 independent experiments are indicated within the columns +/− s.d. Transfectants expressing Exon1B-frameshift-EGFP displayed significantly reduced EGFP expression levels when compared with cells transfected with the construct in which the start codon of uORF4 was deleted (p = 0.049).

To confirm that the uORF4 of the *LST1* exon 1B sequence is responsible for the observed downregulation of protein expression, we mutated the vector containing the exon 1B sequence and deleted the AUG of uORF4 (mutation ATG->AGG, Figure S2C). HeLa cells transiently expressing this construct were analysed by flow cytometry and did not display any significant change in EGFP expression, when compared to cells expressing the empty vector (Figure 3A and D). Similar results were obtained when we assessed the effect of the mutated construct on EGFP expression in HEK-293T cells (Figure S4A and C). In order to further confirm that uORF4 directly regulates protein expression we deleted a single base in the uORF, thereby causing a frameshift mutation that led to a premature stop codon and shortened the uORF (87 bp instead of 117 bp, Figure S2D). Transient expression of this construct in HeLa cells followed by flow cytometry analysis revealed a modest but significant inhibition of EGFP expression, when compared to the empty vector (Figure 3B and E). After assessing the effect of the mutated construct on EGFP expression in HEK-293T cells we obtained comparable results (Figure S4B and D). A quantitative western blot analysis of EGFP expression using these constructs also yielded similar EGFP protein levels (Figure 3C and F). To ensure that the observed effects were not based on fluctuations of transcriptional efficiency, we quantified EGFP transcripts in HeLa and HEK-293T cells transfected either with the unmodified EGFP vector or the fusion constructs employed in this study and found them to yield comparable transcript quantities (Figure S5). The reduced inhibitory potential of the shortened uORF supports our conclusion that uORF4 is responsible for the inhibition of protein expression exerted by the exon 1B sequence, since several studies have found that the inhibitory potential of uORFs increases with their length [22]. However, we cannot rule out the possibility that the altered amino acid sequence of uORF4 may also contribute to the reduced inhibitory potential. Taken together our results lead us to conclude that the uORF4 of exon 1B inhibits protein expression at the translational level.

### Regulation of LST1 protein expression by differential usage of uORF-containing exons

**Figure 4:**
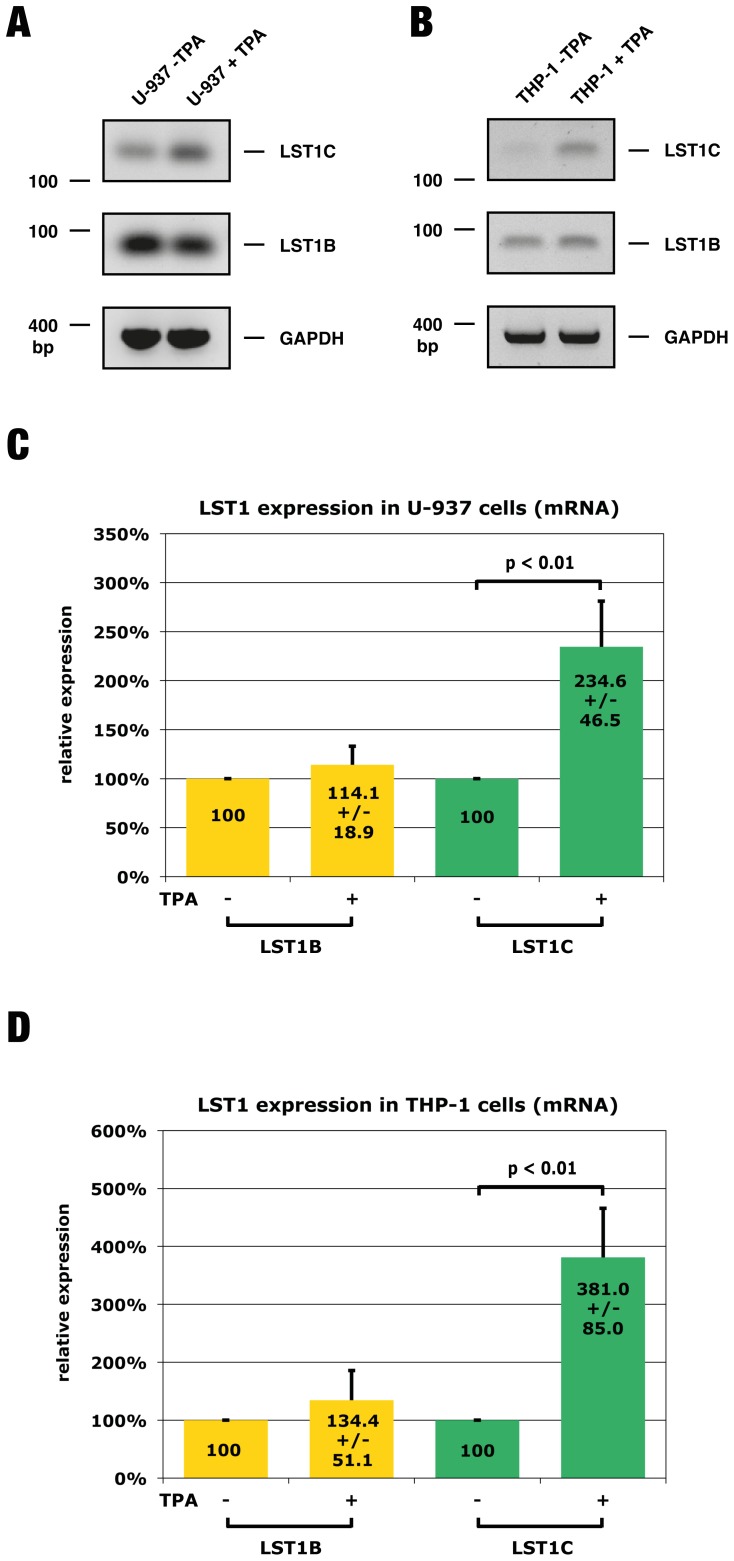
Differential usage of *LST1* uORF-containing exons during monocyte differentiation. Cells from the monocytic cell lines U-937 and THP-1 were prompted to differentiate to macrophages by treatment with 80 nM TPA for 72 h. **(A, B)** *LST1* transcripts containing either exon 1B or 1C were detected by PCR using specific primers and cDNA transcribed from total RNA which was isolated from treated and mock-treated cells. Amplificates for transcripts containing exon 1B and 1C were detected on an agarose gel at 90 and 125 bp, respectively. The amplified GAPDH sequence was detected at 393 bp. **(C, D)** U-937 and THP-1 cells were treated with TPA, RNA was isolated and cDNA was synthesised as described above. The amount of *LST1* transcripts containing either exon 1B (LST1B) or exon 1C (LST1C) was assayed by quantitative PCR. *LST1* transcript expression was normalised using *GAPDH*. A value of 100% was set for mock-treated cells. Mean values from 5 independent experiments are indicated within the columns +/− s.d. In both U-937 and THP-1 cells, treatment with TPA led to a distinct and significant (p = 0.009) increase in the number of transcripts containing exon 1C. For exon 1B containing splice variants only a modest, non-significant (p = 0.094) increase in transcript amounts after TPA treatment of both U-937 and THP-1 cells could be detected.

In a recent study we found LST1 protein levels to be upregulated during monocyte differentiation [23]. In that study, the histiocytic lymphoma cell line U-937, which is of monocytic origin and differentiates upon stimulation with TPA, was used as a model to reproduce the differentiation of monocytes to macrophages. Our finding that the uORF in *LST1* exon 1B downregulates protein expression prompted us to examine whether differential use of exons 1B and 1C may explain the upregulation of LST1 protein expression during monocyte differentiation. In an initial experiment we amplified *LST1* transcripts containing exon 1B or 1C from RNA of undifferentiated or differentiated U-937 cells. As expected, transcripts containing exon 1B predominated in both samples, however in differentiated cells a detectable increase in the amount of transcripts containing the exon 1C sequence was determined (Figure 4A). While the overall amount of transcripts with exon 1C increased upon differentiation, we did not detect a shift in the protein-coding exons 2–5 usage (data not shown). Similar to U-937 cells, the monocytic THP-1 cell line can be prompted to differentiate upon treatment with TPA [24]. We used this cell line to investigate whether the observed shift in *LST1* exon usage is typical for monocyte differentiation. As previously observed in the U-937 cell line, transcription initiation at the *LST1* exon 1C was upregulated in differentiated THP-1 cells (Figure 4B). To confirm these observations we quantified *LST1* transcripts containing either exon 1B or exon 1C via real-time PCR in U-937 and THP-1 cells. In both U-937 and THP-1 cells, differentiation led to a distinct and significant increase in the amount of exon 1C containing transcripts, while variants including exon 1B displayed only a modest increase (Figure 4C and D).

These results lead us to conclude that LST1 protein expression is at least partially regulated by differential usage of uORF-containing exons.

## Discussion

The regulation of gene expression depends on the concurrence of multiple factors at the transcriptional and translational level. In the present study we questioned whether the expression of the MHC class III gene *LST1* is regulated at the translational level via differential usage of uORF-containing exons in transcripts. First, we characterized the 5′-UTR of *LST1* and found it to contain several uORFs displaying various lengths (Figure 1A–B). Reporter assays revealed the exon 1B sequence to negatively regulate protein expression, while the exon 1C sequence (lacking an uORF) had no effect (Figure 2). Using reporter constructs containing mutated exon 1B sequences we were able to demonstrate that the uORF in exon 1B is responsible for this inhibition (Figure 3). Downregulation of translation due to miRNA binding to the AUG of uORF4 as proposed by [25] seems unlikely since none of the listed miRs could interact with this particular AUG. Since similar levels of exon 1B-EGFP and exon 1C-EGFP transcripts were observed in transfectants (Figure S5), it is also unlikely that uORF4 induced nonsense-mediated mRNA decay is responsible for reduced protein levels.

These results indicate that LST1 expression is tightly regulated at the translational level and that basal expression underlies negative regulation by the uORF4 of exon 1B. The strength of translational inhibition is likely to be determined by a number of features within this uORF. First, as discussed in the results section, the above-average uORF length and proximity to the main ORF have been previously linked to a greater translational inhibition. Second, uORF4 features a Kozak sequence of moderate strength, while the main *LST1* ORF exhibits a weak Kozak sequence, a setting that is unlikely to promote leaky ribosome scanning. Third, specific sequence characteristics have been associated with high-efficiency reinitiation after translation of an uORF: an A/U-rich codon preceding the stop codon and an A/U-rich sequence in the 10 bp immediately downstream of this uORF stop codon [26]. The *LST1* uORF4 features a proline-encoding codon (CCC) preceding the stop codon, and in the immediate downstream sequence only 5 out of 10 bases are A/U. Therefore, the reinitiation efficiency is predicted to be moderate at best. Taken together, our data and the characteristics of uORF4 indicate that LST1 protein expression underlies tight translational regulation.

Having established the negative effect of uORF4 on LST1 expression, we tested whether differential use of exons 1B and 1C in transcripts may be responsible for increased LST1 expression during monocyte differentiation. We found a distinct shift towards usage of exon 1C, which correlated with increased protein levels (Figure 4). The increase in LST1 molecules upon differentiation would affect the immunoregulatory properties of the cells. Two functions have been assigned to full-length LST1 proteins: transmembrane adaptor protein (TRAP) involved in the inhibition of yet to be identified signalling pathways [27], [28] and induction of tunneling nanotubes (TNT) allowing direct long distance communication and transport between cells [12]. Both tasks could be enhanced by the increase in LST1 protein levels. It is noteworthy that we focussed on the effect of the exon 1B and 1C sequences on protein expression mainly because initiation of transcription at exons 1A and 1D is very rare [8] and initiation at exon 1E predominates only in fetal tissue [29]. The presence of several uORFs in exon 1E hints that LST1 expression might be strongly repressed in fetal tissues. This interesting possibility could be addressed in future studies. Furthermore, it is also noteworthy that all *LST1* transcripts include uORF7 due to the AUG at the beginning of exon 2. The strength of its Kozak sequence depends on the preceding exon 1 sequence. When either exon 1B or 1C are located upstream, the resulting Kozak sequence is very weak. This explains our results indicating that uORF7 has no effect on protein expression, as the reporter construct containing both exon 1C and uORF7 was expressed at levels comparable to those of the empty vector.

A considerable percentage of human genes are regulated by alternative untranslated regions (for a review see [30]) and in many cases regulation is based on the presence of uORFs. Yet until now, functional activity has been validated only for a few uORFs [4], [31]. In this study we have shown that LST1 protein expression is regulated by alternative transcription initiation in exons containing or lacking an uORF. For several genes like *AXIN2*, *BRCA1* and *MDM2* alternative transcription initiation sites have been shown to result in uORF-based translational repression [32], [33], [34]. However, in these cases transcription initiation was regulated by alternative promoters. The *LST1* gene lacks typical TATA promoter sequences and transcription factor binding sites. The region downstream of exon 1B up to exon 1C contains potential binding sites for transcription factors and was identified as the sequence with highest promoter activity in reporter assays [8]. The authors also identified sequences just upstream of exon 1B with protein binding sites but the respective nuclear factors could not be identified. These results may explain the abundance of *LST1* transcripts initiated at exon 1B. It appears unlikely that alternative transcription initiation is controlled by alternative promoters. Instead, transcription initiation may be determined by binding of a yet unidentified set of transcription factors. While we have shown that the presence of uORF4 in *LST1* transcripts leads to translational repression we do not know its physiological role as exon 1B transcripts are the major *LST1* transcripts detected in cells. One could speculate that the encoded peptide plays a role by acting in a cis or trans manner [35]. The uORF4 peptide sequence is conserved in pan, gorilla and pongo, but differences in the amino acid sequence increase with evolutionary distance. Another possibility is that timed or local uORF4 bypass may allow quick LST1 protein expression. Finally, we cannot exclude the possibility that several mechanisms for translational control of LST1 expression are in place. Upstream ORFs are not static repressors of translational efficiency but rather components of dynamic systems for gene expression regulation. Translation initiation at a specific uORF can be upregulated by specific proteins or the downregulation of miRNAs [36], [25] or completely bypassed under certain stress conditions [31], [35], [37].

In summary, in the present study we describe to our knowledge the first mechanism for the regulation of LST1 protein levels. We consider this mechanism to be clinically relevant, since high LST1 mRNA levels have been associated in previous studies with inflammatory pathologies.

## Supporting Information

Figure S1 The uORF in *LST1* exon 1B is evolutionary conserved. A) Comparison of the *LST1* exon 1B-2 sequence with several homologues, beginning upstream of uORF4 (red, bold) and ending at the start codon of the main ORF (black, bold). The sequence is highly conserved in pan troglodytes (sequence accession number XM_003950777.1, 100% identity) and macaca mulatta (NW 001116486.1, 95% identity). Furthermore, the sequence is partly conserved in sus scrofa (NC_010449.4, 76% identity, 16 gaps) and bos taurus (NC_007324.5, 74% identity, 8 gaps). It is noteworthy that in sus scrofa and bos taurus the stop codon of uORF4 is not conserved. **B)** Comparison of the amino acid sequence encoded by uORF4 with several homologues. The sequence is highly conserved in pan troglodytes (100% identity) and macaca mulatta (89% identity). However, in sus scrofa (67% identity, 5 gaps) and bos taurus (39% identity, 2 gaps) the sequence is only partly conserved. Note that in bos taurus uORF4 is longer than in homo sapiens (45 versus 38 amino acids) and that in sus scrofa due to the lack of a stop codon uORF4 is in frame with the main ORF, therefore constituting an uAUG rather than an uORF.
(TIF)

Figure S2 Expression vectors used in this study. Overview of the expression vectors used in this study. The sequences shown were cloned into the multiple cloning site (MCS) of the pEGFP-N1 vector; restriction enzyme cutting sites used for cloning are displayed in bold. The exon sequences and the MCS of pEGFP-N1 are labelled, the start codon of uORF4 and of the main EGFP ORF are highlighted in grey. The amino acids encoded by uORF4 and by the main ORF are indicated beneath the nucleic acid sequence. Note that all constructs contain an ATG in exon 2 and encode ORF7, this exon is present in all LST1 transcripts, therefore its usage is not regulated by the mechanisms investigated in this study. The cloning of the exon 1B-2 and 1C-2 sequences into the pEGFP-N1 vector resulted in the deletion of 14 nucleotides located upstream of the *LST1* main ORF. In these expression vectors the EGFP ORF is preceded by 19 nucleotides from the pEGFP-N1 MCS. The expression vectors Exon1B-EGFP, Exon1B-AUGdel-EGFP and Exon1B-frameshift-EGFP include a 23 bp long sequence assigned to intron 1A, this is due to the ambiguity of published exon 1B sequences. The sequence shown here is labelled according to [10], however a second study reports the exon 1B sequence to begin 23 bp upstream [8].
(TIF)

Figure S3 The *LST1* exon 1B sequence inhibits protein expression. HeLa cells were cotransfected with in vitro transcribed RNA (ivt RNA) from expression constructs encoding the red fluorescent protein mCherry and either an EGFP expression vector or an Exon1B-EGFP fusion construct. **(A)** Flow cytometry analysis of EGFP intensity in HeLa transfectants expressing Exon1B-EGFP ivt RNA (yellow line) or the EGFP ivt RNA (blue line). Untransfected cells are displayed as a solid grey curve. The mCherry expression was used to gate and select positive transfectants, which were analysed for the intensity of EGFP expression. **(B)** Quantitative flow cytometry analysis of EGFP expression in HeLa transfectants. The analysis of EGFP expression was performed as described in (A) and the mean fluorescence intensity was quantified. A value of 100% was set for cells transfected with ivt RNA from the unmodified EGFP vector. Mean values from 3 independent experiments are indicated within the columns +/− s.d. Transfectants expressing Exon1B-EGFP displayed significantly reduced EGFP expression levels when compared with cells transfected with ivt EGFP RNA (p = 0.049).
(TIF)

Figure S4 The uORF in *LST1* exon 1B inhibits protein expression. HEK-293T cells were cotransfected with expression constructs encoding the red fluorescent protein mCherry and either an EGFP expression vector, Exon1B-EGFP, Exon1B-AUGdel-EGFP or Exon1B-frameshift-EGFP fusion constructs. **(A, B)** Flow cytometry analysis of EGFP intensity in HEK-293T transfectants expressing Exon1B-EGFP (yellow line), Exon1B-AUGdel-EGFP, Exon1B-frameshift-EGFP (green line in A and B, respectively) or the unmodified EGFP vector (blue line). Untransfected cells are displayed as a solid grey curve. The mCherry expression was used to gate and select positive transfectants, which were analysed for the intensity of EGFP expression. **(C, D)** Quantitative flow cytometry analysis of EGFP expression in HEK-293T transfectants. The analysis of EGFP expression was performed as described in (A, B) and the mean fluorescence intensity was quantified. A value of 100% was set for cells transfected with the unmodified EGFP vector. Mean values from 5 independent experiments are indicated within the columns +/− s.d. Transfectants expressing Exon1B-EGFP displayed significantly reduced EGFP levels when compared with cells transfected with the empty vector (p = 0.009). The expression of the Exon1B-AUGdel-EGFP vector, in which the start codon of the uORF was mutated, was comparable to the expression of the unmodified construct. Expression of the Exon1B-frameshift-EGFP construct, in which a frameshift mutation shortens the uORF, was significantly stronger when compared to cells transfected with the Exon1B-EGFP vector (p = 0.049), but still considerably weaker than the expression of the unmodified vector.
(TIF)

Figure S5 Quantification of *EGFP* transcript expression in transfectants using qPCR. HeLa **(A)** and HEK-293T **(B)** cells were transfected with either an EGFP expression vector, Exon1B-EGFP, Exon1C-EGFP, Exon1B-AUGdel-EGFP or Exon1B-frameshift-EGFP fusion constructs. Total RNA was isolated from transfectants and cDNA was synthesised. The amount of *EGFP* transcripts was assayed by quantitative PCR. *EGFP* transcript expression was first normalised for *GAPDH* and then for expression of the neomycin resistance gene. The later was performed to compensate for fluctuations in transfection efficiency, as the neomycin resistance gene is present in all expression vectors. A value of 100% was set for cells transfected with the unmodified EGFP vector. Mean values from 3 independent experiments are indicated within the columns +/− s.d. Both HeLa and HEK-293T cells transfected with either the unmodified EGFP expression vector or any of the fusion constructs employed in this study displayed comparable *EGFP* transcript levels.
(TIF)
