# Supplementary figures and images for: An Upstream Open Reading Frame Regulates LST1 Expression during Monocyte Differentiation
Source: PLoS One. 2014 May 9;9(5):e96245. doi: 10.1371/journal.pone.0096245 (PMC4015914; doi:10.1371/journal.pone.0096245)

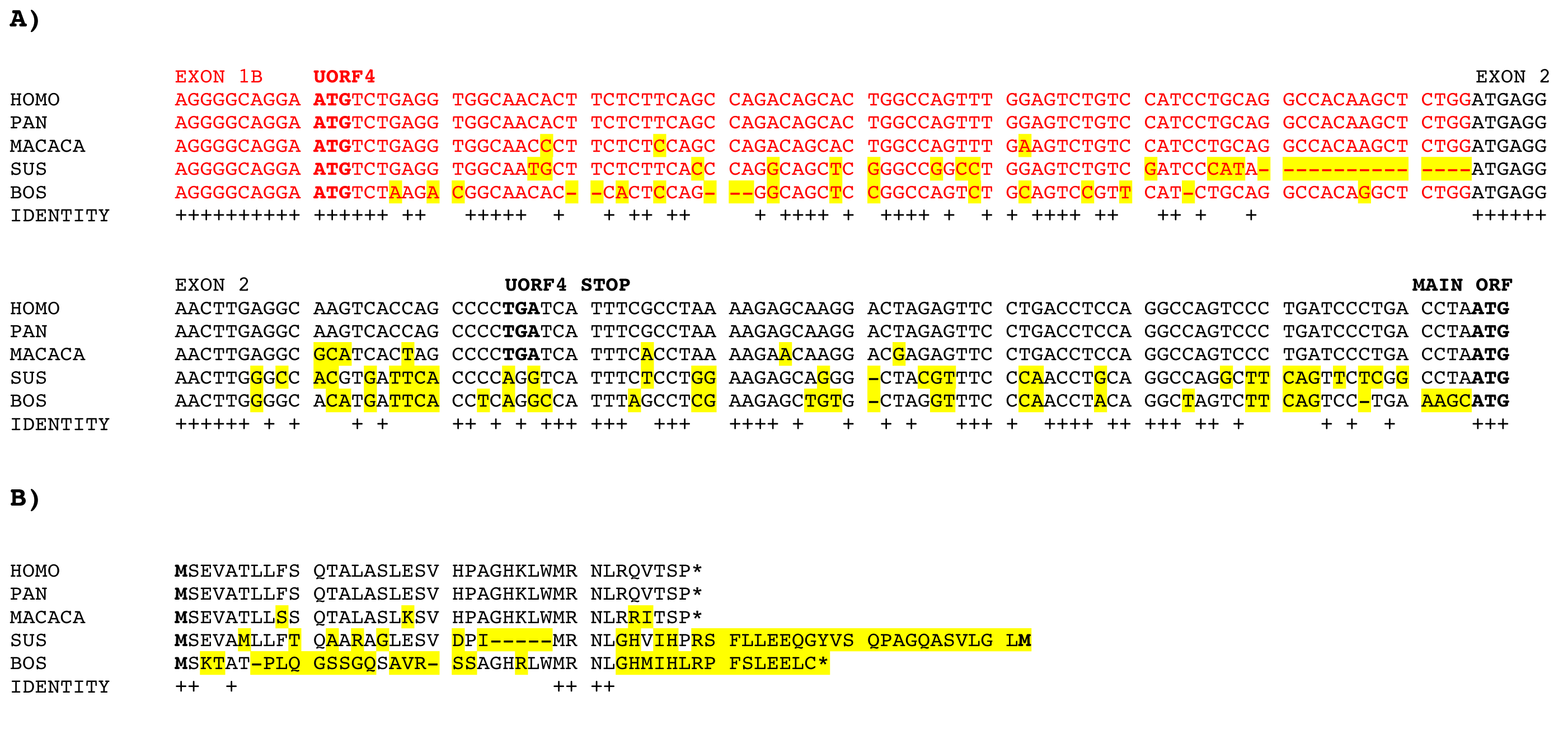

Supplement: Figure S1 — The uORF in LST1 exon 1B is evolutionary conserved. A) Comparison of the LST1 exon 1B-2 sequence with several homologues, beginning upstream of uORF4 (red, bold) and ending at the start codon of the main ORF (black, bold). The sequence is highly conserved in pan troglodytes (sequence accession number XM_003950777.1, 100% identity) and macaca mulatta (NW 001116486.1, 95% identity). Furthermore, the sequence is partly conserved in sus scrofa (NC_010449.4, 76% identity, 16 gaps) and bos taurus (NC_007324.5, 74% identity, 8 gaps). It is noteworthy that in sus scrofa and bos taurus the stop codon of uORF4 is not conserved. B) Comparison of the amino acid sequence encoded by uORF4 with several homologues. The sequence is highly conserved in pan troglodytes (100% identity) and macaca mulatta (89% identity). However, in sus scrofa (67% identity, 5 gaps) and bos taurus (39% identity, 2 gaps) the sequence is only partly conserved. Note that in bos taurus uORF4 is longer than in homo sapiens (45 versus 38 amino acids) and that in sus scrofa due to the lack of a stop codon uORF4 is in frame with the main ORF, therefore constituting an uAUG rather than an uORF. (TIF) [file pone.0096245.s001.tif]

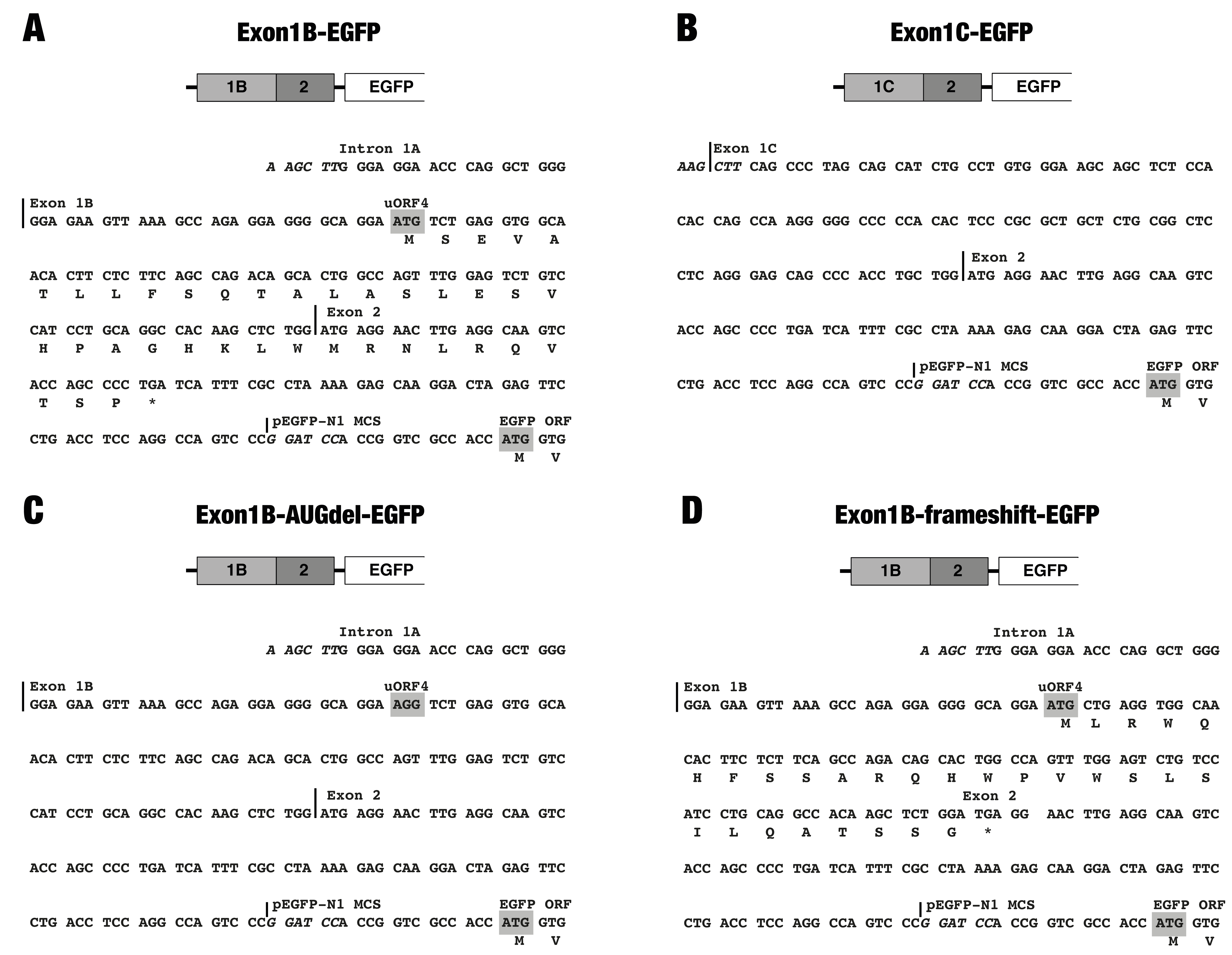

Supplement: Figure S2 — Expression vectors used in this study. Overview of the expression vectors used in this study. The sequences shown were cloned into the multiple cloning site (MCS) of the pEGFP-N1 vector; restriction enzyme cutting sites used for cloning are displayed in bold. The exon sequences and the MCS of pEGFP-N1 are labelled, the start codon of uORF4 and of the main EGFP ORF are highlighted in grey. The amino acids encoded by uORF4 and by the main ORF are indicated beneath the nucleic acid sequence. Note that all constructs contain an ATG in exon 2 and encode ORF7, this exon is present in all LST1 transcripts, therefore its usage is not regulated by the mechanisms investigated in this study. The cloning of the exon 1B-2 and 1C-2 sequences into the pEGFP-N1 vector resulted in the deletion of 14 nucleotides located upstream of the LST1 main ORF. In these expression vectors the EGFP ORF is preceded by 19 nucleotides from the pEGFP-N1 MCS. The expression vectors Exon1B-EGFP, Exon1B-AUGdel-EGFP and Exon1B-frameshift-EGFP include a 23 bp long sequence assigned to intron 1A, this is due to the ambiguity of published exon 1B sequences. The sequence shown here is labelled according to [10], however a second study reports the exon 1B sequence to begin 23 bp upstream [8]. (TIF) [file pone.0096245.s002.tif]

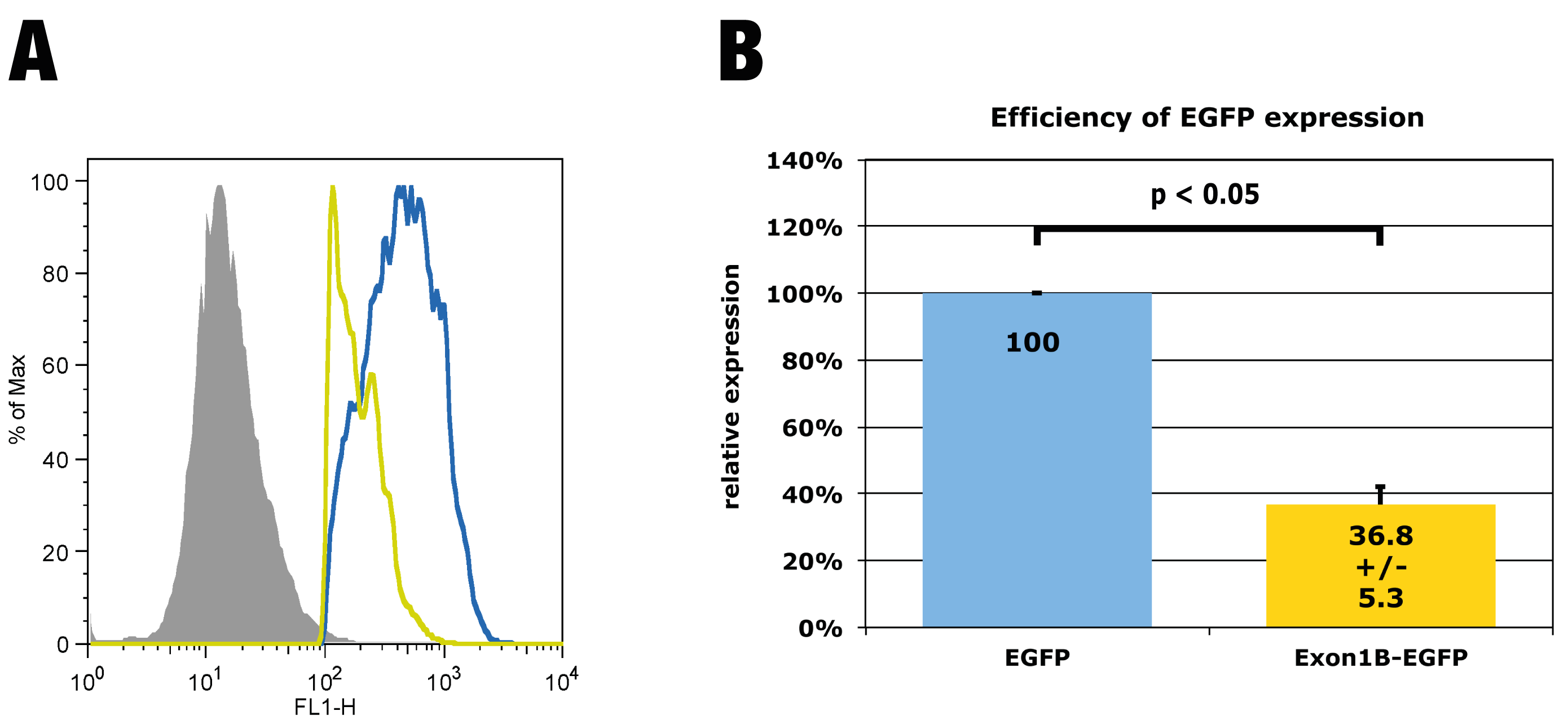

Supplement: Figure S3 — The LST1 exon 1B sequence inhibits protein expression. HeLa cells were cotransfected with in vitro transcribed RNA (ivt RNA) from expression constructs encoding the red fluorescent protein mCherry and either an EGFP expression vector or an Exon1B-EGFP fusion construct. (A) Flow cytometry analysis of EGFP intensity in HeLa transfectants expressing Exon1B-EGFP ivt RNA (yellow line) or the EGFP ivt RNA (blue line). Untransfected cells are displayed as a solid grey curve. The mCherry expression was used to gate and select positive transfectants, which were analysed for the intensity of EGFP expression. (B) Quantitative flow cytometry analysis of EGFP expression in HeLa transfectants. The analysis of EGFP expression was performed as described in (A) and the mean fluorescence intensity was quantified. A value of 100% was set for cells transfected with ivt RNA from the unmodified EGFP vector. Mean values from 3 independent experiments are indicated within the columns +/− s.d. Transfectants expressing Exon1B-EGFP displayed significantly reduced EGFP expression levels when compared with cells transfected with ivt EGFP RNA (p = 0.049). (TIF) [file pone.0096245.s003.tif]

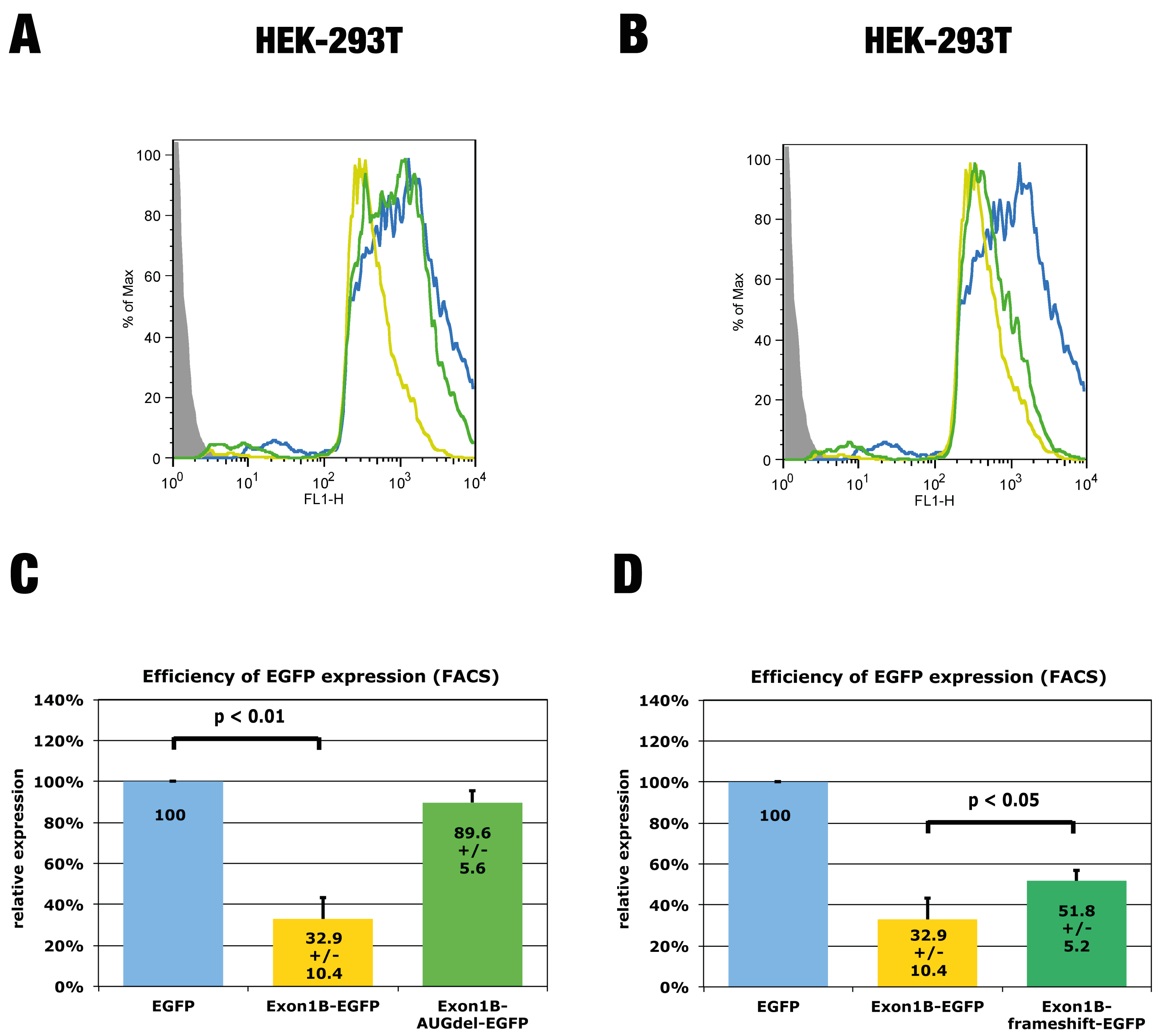

Supplement: Figure S4 — The uORF in LST1 exon 1B inhibits protein expression. HEK-293T cells were cotransfected with expression constructs encoding the red fluorescent protein mCherry and either an EGFP expression vector, Exon1B-EGFP, Exon1B-AUGdel-EGFP or Exon1B-frameshift-EGFP fusion constructs. (A, B) Flow cytometry analysis of EGFP intensity in HEK-293T transfectants expressing Exon1B-EGFP (yellow line), Exon1B-AUGdel-EGFP, Exon1B-frameshift-EGFP (green line in A and B, respectively) or the unmodified EGFP vector (blue line). Untransfected cells are displayed as a solid grey curve. The mCherry expression was used to gate and select positive transfectants, which were analysed for the intensity of EGFP expression. (C, D) Quantitative flow cytometry analysis of EGFP expression in HEK-293T transfectants. The analysis of EGFP expression was performed as described in (A, B) and the mean fluorescence intensity was quantified. A value of 100% was set for cells transfected with the unmodified EGFP vector. Mean values from 5 independent experiments are indicated within the columns +/− s.d. Transfectants expressing Exon1B-EGFP displayed significantly reduced EGFP levels when compared with cells transfected with the empty vector (p = 0.009). The expression of the Exon1B-AUGdel-EGFP vector, in which the start codon of the uORF was mutated, was comparable to the expression of the unmodified construct. Expression of the Exon1B-frameshift-EGFP construct, in which a frameshift mutation shortens the uORF, was significantly stronger when compared to cells transfected with the Exon1B-EGFP vector (p = 0.049), but still considerably weaker than the expression of the unmodified vector. (TIF) [file pone.0096245.s004.tif]

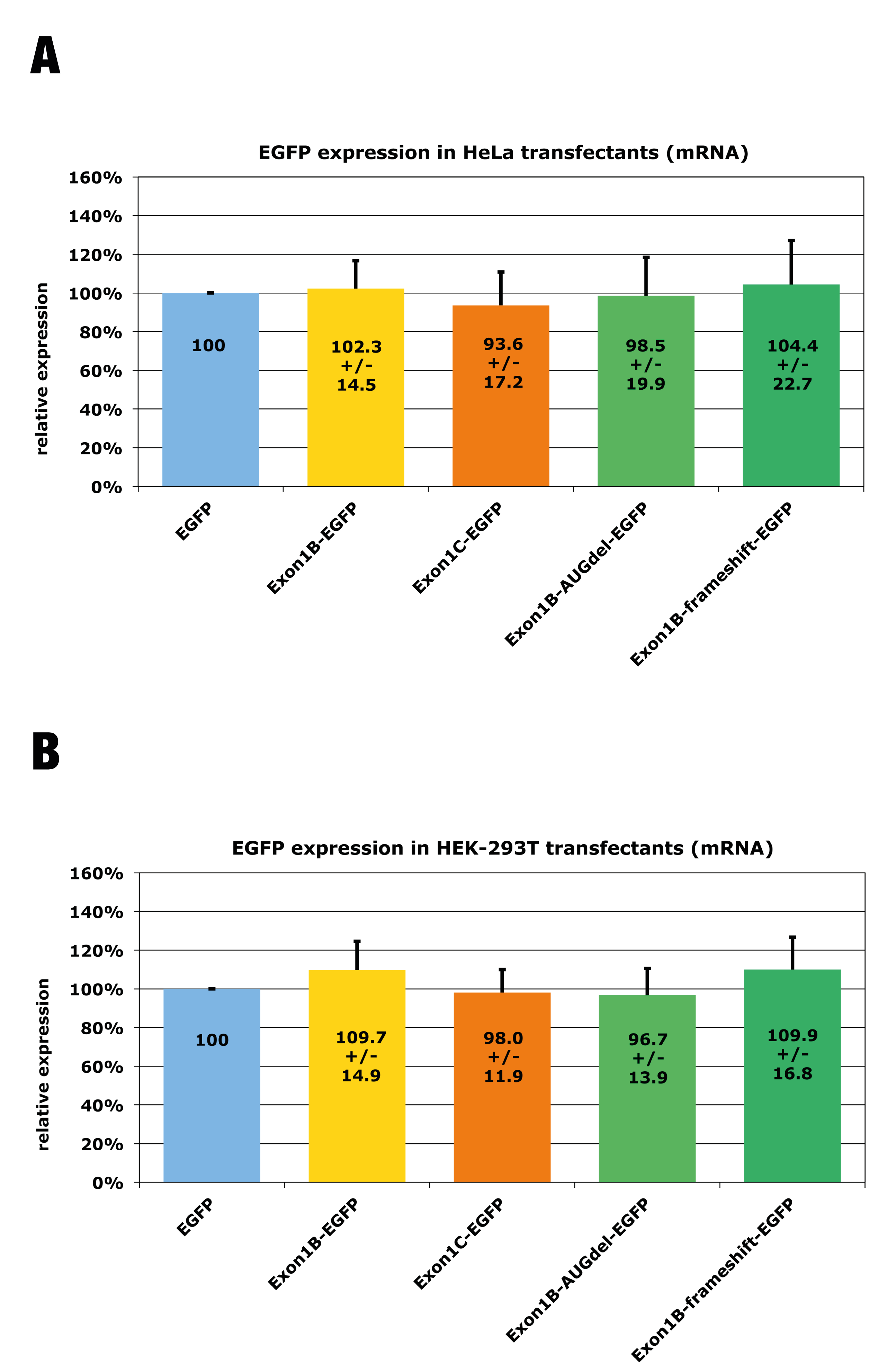

Supplement: Figure S5 — Quantification of EGFP transcript expression in transfectants using qPCR. HeLa (A) and HEK-293T (B) cells were transfected with either an EGFP expression vector, Exon1B-EGFP, Exon1C-EGFP, Exon1B-AUGdel-EGFP or Exon1B-frameshift-EGFP fusion constructs. Total RNA was isolated from transfectants and cDNA was synthesised. The amount of EGFP transcripts was assayed by quantitative PCR. EGFP transcript expression was first normalised for GAPDH and then for expression of the neomycin resistance gene. The later was performed to compensate for fluctuations in transfection efficiency, as the neomycin resistance gene is present in all expression vectors. A value of 100% was set for cells transfected with the unmodified EGFP vector. Mean values from 3 independent experiments are indicated within the columns +/− s.d. Both HeLa and HEK-293T cells transfected with either the unmodified EGFP expression vector or any of the fusion constructs employed in this study displayed comparable EGFP transcript levels. (TIF) [file pone.0096245.s005.tif]
